# Sexual dimorphism of detrusor function demonstrated by urodynamic studies in rhesus macaques

**DOI:** 10.1038/s41598-020-73016-0

**Published:** 2020-09-30

**Authors:** Natalia P. Biscola, Kari L. Christe, Ephron S. Rosenzweig, Mark H. Tuszynski, Leif A. Havton

**Affiliations:** 1 Department of Neurology, Icahn School of Medicine at Mount Sinai, New York, NY USA; 2 California National Primate Research Center, UC Davis, Davis, CA USA; 3 Department of Neurosciences, UC San Diego, La Jolla, CA USA; 4 Veterans Administration Medical Center, La Jolla, CA USA; 5 Departments of Neurology and Neuroscience, Icahn School of Medicine At Mount Sinai, 1468 Madison Avenue, New York, NY 10029 USA; 6 RR&D National Center for the Medical Consequences of Spinal Cord Injury and Neurology Service, James J. Peters VA Medical Center, Bronx, NY USA

**Keywords:** Neurology, Urology

## Abstract

The lower urinary tract (LUT) and micturition reflexes are sexually dimorphic across mammals. Sex as a biological variable is also of critical importance for the development and translation of new medical treatments and therapeutics interventions affecting pelvic organs, including the LUT. However, studies of LUT function with comparisons between the sexes have remained sparse, especially for larger mammals. Detrusor function was investigated by filling cystometry and pressure flow studies in 16 male and 22 female rhesus macaques. By filling cystometry, male subjects exhibited a significantly larger bladder capacity and compliance compared to females. Pressure flow studies showed a significantly higher bladder pressure at voiding onset, peak pressure, and elevation in detrusor-activated bladder pressure from the end of bladder filling to peak pressure in the male subjects. The activation of reflex micturition, with associated detrusor contractions, resulted in voiding in a significantly larger proportion of female compared to male subjects. A higher urethral outlet resistance is suggested in the male subjects. We conclude that sexual dimorphism of detrusor function is prominent in rhesus macaques, shares many features with the human, and merits consideration in translational and pre-clinical research studies of micturition and LUT function in non-human primates.

## Introduction

The lower urinary tract (LUT) shows two functional states in mammals, storage and elimination, and its control is regulated by the coordinated actions of the autonomic and somatic motor nervous systems^1,2^. The detrusor muscle is relaxed and maintains a compliant reservoir during the bladder storage phase, whereas willful voiding takes place during active detrusor contractions to increase the intraluminal pressure, overcome urethral resistance, and promote urine flow^3,4^. Micturition is sexually dimorphic, in part due to anatomical differences between males and females in the structural relationships between the LUT and adjacent pelvic organs^5^. Sex as a biological variable is well recognized and represents an important consideration for research studies in both humans and animal models^6^. However, direct comparisons of micturition reflexes and voiding function between male and female subjects have been sparse, especially for studies in large mammals.

Non-human primate research models continue to play a critical role for the advancement of promising new medical treatment strategies towards clinical use^7,8^. The rhesus macaque was identified early as a favored animal model in urologic research^9^ and more recent urodynamic studies in rhesus macaques have shown translational research utility and great value in studies of the effects of anesthesia, novel pharmacological agents, aging, and emerging strategies for neuromodulation of reflex micturition^10–15^. However, urodynamic studies in rhesus macaques have been performed only in female animals, in part due to the marked technical challenges of placing transurethral catheters in male primates. Therefore, no comprehensive urodynamic data sets from male rhesus macaques are available, and no comparative studies on voiding function in males and females have been performed in primate models. We hypothesized that rhesus macaques show sexual dimorphism in both passive and active detrusor contractility as well as in voiding function.

In the present study, we refined a strategy for the placement of multi-lumen transurethral catheters in male rhesus macaques, taking into consideration both anatomical features of the male urethra and differential muscle-relaxation properties of anesthetic agents. Next, we performed comprehensive urodynamic studies of reflex micturition in both male and female animals to characterize possible sex-related differences in LUT function with regards to both passive properties of the detrusor and pressure flow features during active voiding.

## Results

Comprehensive urodynamic studies were performed in adult male (n = 16) and female (n = 22) rhesus macaques.

### Filling cystometry

**Figure 1:**
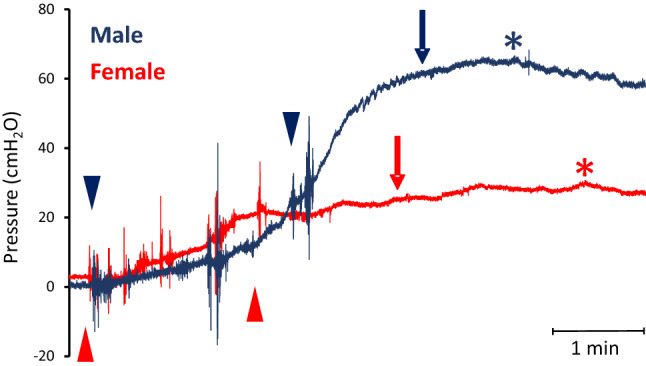
Reflex micturition is evoked in both male and female rhesus macaques and demonstrated by urodynamic studies. Filling cystometry and pressure flow studies result in characteristic bladder pressure recordings. Following emptying of the bladder, saline is infused to raise bladder pressure about 25 cmH_2_O above baseline. Both the male and female recordings show a gradual increase in bladder pressure during the filling phase, followed by a delayed voiding phase. A representative trace is presented from a male (blue) and female (red) subject. The onset and end of the bladder filling is indicated by arrow heads, the onset of voiding by an arrow, and peak bladder pressure by an asterisk for each subject. Note that the bladder pressure at onset of voiding and the peak bladder pressure are markedly higher in the male compared to the female subject.

**Figure 2:**
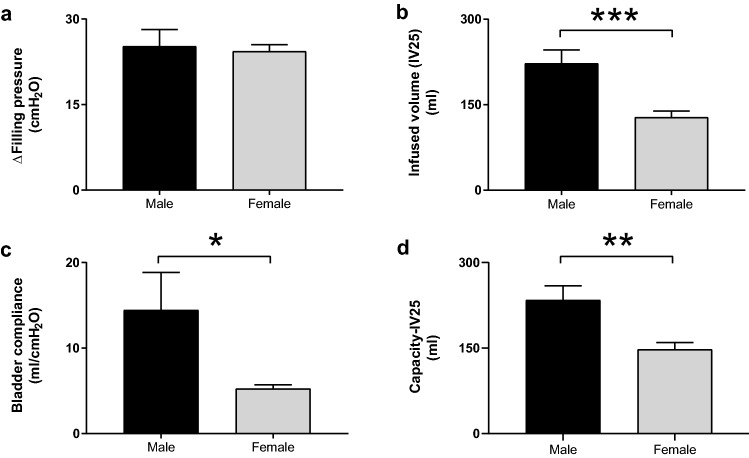
Filling cystometry show sexual dimorphism in rhesus macaques. Male (n = 16) and female (n = 22) rhesus macaques underwent infusion of saline into the bladder using transurethral multi-lumen catheter. The bladder pressure was similarly raised and not different in male and female rhesus macaques (**a**). The infused volume to raise the bladder pressure 25 cmH_2_O (IV25), bladder compliance (Bcomp), and Capacity-IV25 were significantly higher in the male subjects compared to the females (**b**–**d**).

In both male and female rhesus macaques, the fill port of the transurethral catheter was used for saline infusion to raise the bladder pressure to a targeted 25 cmH_2_O above the baseline pressure of an emptied bladder (Figs. 1 and 2). Saline infusion raised the bladder pressure 25.2 ± 2.9 cmH_2_O in males (n = 16) and 24.4 ± 1.1 cmH_2_O in the female subjects (n = 22) (Fig. 2a). There was no statistical difference in the infusion-induced increase in bladder pressure between the two cohorts. The saline infusion studies showed a significantly larger infused volume to raise the bladder pressure 25 cmH_2_O (IV25) of 222.6 ± 23.6 ml and a higher bladder compliance (Bcomp) of 14.5 ± 4.4 ml/cmH_2_O in the male subjects (n = 16) compared to the corresponding IV25 of 128.2 ± 10.6 ml (*p* = 0.0003) and Bcomp of 5.2 ± 0.5 ml/cmH_2_O in the females (*p* = 0.01) (n = 22) (Fig. 2b,c). The combined voided volume and PVR after the partial bladder filling to raise the pressure 25 cmH2O, Capacity-IV25, was significantly larger in males at 234.4 ± 24.8 ml (n = 16) compared to females at 147.7 ± 11.9 ml (n = 22) (*p* = 0.0016) (Fig. 2d).

### Reflex pressure flow studies

**Figure 3:**
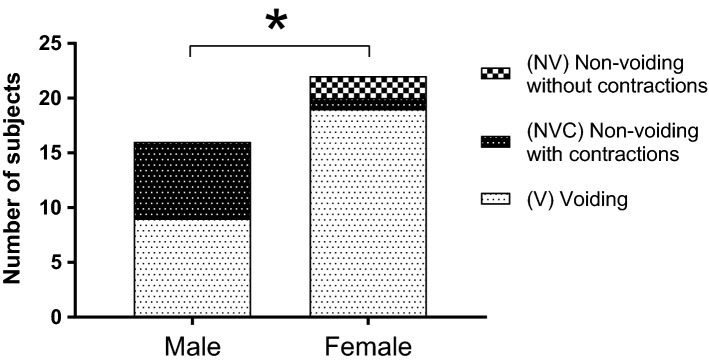
Reflex micturition shows sexual dimorphism in rhesus macaques. Partial filling of the bladder was followed by assessments for delayed reflex micturition and voiding in male (n = 16) and female (n = 22) rhesus macaques. The micturition reflex was activated in 100% (16/16) of male and 90.9% (20/22) of female subjects. No voiding (NV) with absence of reflex detrusor contractions was shown in 2 female subjects. Non-voiding contractions (NVC) with bladder pressure elevation were present in 1 female subject. Detection of NVC was present in 43.8% (7/16) of the male subjects. Voiding (V) was present in a significantly lower proportion of 56.2% (9/16) of the male subjects compared to 86.4% (19/22) of the females (*p* < 0.05).

Delayed reflex detrusor contractions and voiding typically followed the partial filling of the bladder (Figs. 1 and 3). Activation of the micturition reflex with detrusor contractions was detected in 100% (16/16) of male and 90.9% (20/22) of female subjects (Fig. 3). Absence of reflex detrusor contractions was demonstrated in 2 female subjects. However, detrusor contractions resulting in voiding was present in a significantly lower proportion of 56.2% (9/16) of the male subjects compared to 86.4% (19/22) of the females (*p* < 0.05) (Fig. 3).

**Figure 4:**
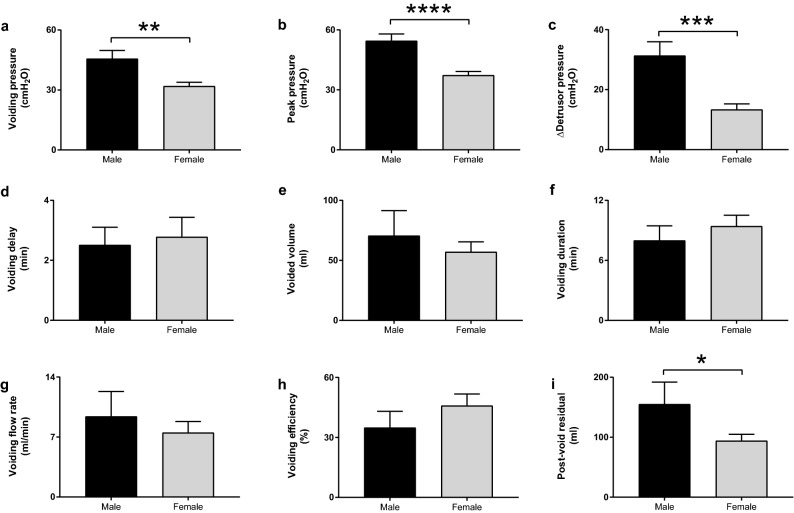
Pressure flow studies show sexual dimorphism in rhesus macaques. Studies of subjects with demonstrated micturition reflex activation and voiding were performed in male (n = 9) and female (n = 19) rhesus macaques. The bladder pressure at voiding onset (Pvoid) (**a**), peak pressure (Ppeak) (**b**), and change in bladder pressure from end of bladder infusion to peak pressure (P∆detr) (**c**) were significantly higher in the males compared to female subjects. There was no difference between the male and female subjects with regards to delay from end of bladder infusion to the onset of voiding (Vdelay) (**d**), voided volume (Vv) (**e**), duration of voiding (Vdur) (**f**), flow rate of voiding (Vflow) (**g**), and voiding efficiency (VE) (**h**). The post-void residual volume (PVR) was larger in males compared to the female subjects (**i**).

The next set of pressure flow comparisons were made between subjects with demonstrated reflex voiding and included both male (n = 9) and female (n = 19) subjects (Fig. 4). Reflex voiding onset was initiated at a significantly higher bladder pressure (Pvoid) of 45.6 ± 4.2 cmH_2_O in the male subjects compared to the corresponding Pvoid onset of 31.9 ± 2.0 cmH_2_O in females (*p* = 0.002) (Fig. 4a). During the active reflex detrusor contractions and voiding, the peak bladder pressure (Ppeak) was significantly higher in the males at 54.5 ± 3.5 cmH_2_O compared to the corresponding Ppeak of 37.2 ± 1.9 cmH_2_O in the female subjects (*p* < 0.0001) (Fig. 4b). The increase in bladder pressure from P∆fill to Ppeak as a marker for bladder pressure augmentation from reflex detrusor contractions (P∆detr) was similarly significantly higher in the males subjects at 31.3 ± 4.6 cmH_2_O compared to the corresponding pressure elevations of 13.4 ± 1.9 cmH_2_O in females (*p* = 0.0002) (Fig. 4c). The delay of voiding after the completion of partial bladder filling (Vdelay) was 2.51 ± 0.59 min in the male subjects and not different from a Vdelay of 2.78 ± 0.66 in females (Fig. 4d). The voiding volume (Vv) was 70.6 ± 20.9 ml and not different from a Vv of 57.0 ± 8.5 ml in females (Fig. 4e). The voiding duration (Vdur) was 8.0 ± 1.5 min in males and not different from Vdur of 9.4 ± 1.1 min in the female subjects (Fig. 4f). The flow of bladder contents during voiding (Vflow) was 9.4 ± 2.9 ml/min in males and not different from Vflow of 7.5 ± 1.3 ml/min in females (Fig. 4g). The voiding efficiency (VE) was 34.9 ± 8.2% and not different from the VE of 45.9 ± 5.8% in females (Fig. 4h). The post-voiding residual volume (PVR) was 155 ± 37 ml in males and significantly larger than the corresponding PVR of 94 ± 11 ml in females (*p* < 0.05) (Fig. 4i).

### Voiding versus non-voiding detrusor contractions

**Figure 5:**
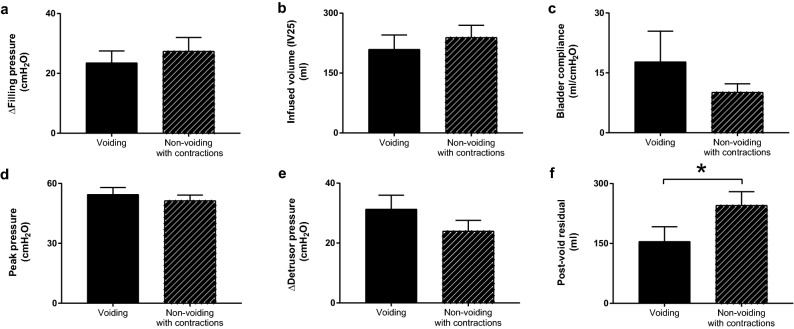
Voiding and non-voiding detrusor contractions show similar features in male rhesus macaques. Passive and active detrusor properties in male rhesus macaques were compared between subjects with voiding (n = 9) and with non-voiding with contractions (NVC) (n = 7). There was no difference between the two groups with regards to bladder pressure at the end of bladder filling (PΔfill) (**a**), infused volume to raise the bladder pressure 25 cmH_2_O (IV25) (**b**), bladder compliance (Bcomp) (**c**), peak pressure (Ppeak) (**d**), and change in bladder pressure from end of bladder infusion to peak pressure (PΔdetr) (**e**). The post-void residual volume (PVR) was significantly higher in the NVC group compared to the male subjects with demonstrated voiding (**f**).

In an attempt to understand potential mechanisms for whether voiding occurs or not following activation of the micturition reflex in male rhesus macaques, a comparison was made between male subjects with and without voiding contractions with regards to key features of filling cystometry and pressure flow studies. Pfill as a sensory stimulus to activate the micturition reflex was 24.4 ± 4.7 cmH_2_O for males with voiding and not different from a Pfill of 29.4 ± 4.0 cmH_2_O in males with non-voiding contractions (NVC) (Fig. 5a). The passive detrusor properties of infused volume to raise the bladder pressure 25 cmH_2_O (IV25) was 210 ± 36 ml and Bcomp was 17.8 ± 7.6 ml/cmH_2_O in males with voiding and not different from a IV25 of 239 ± 30 ml and Bcomp of 10.2 ± 2.1 ml/cmH_2_O in males with NVC, respectively (Fig. 5b,c). In subsequent pressure flow studies, Ppeak was 54.5 ± 3.5 cmH_2_O and P∆detr was 31.3 ± 4.6 cmH_2_O in males with voiding and not different from Ppeak of 51.4 ± 2.8 cmH_2_O and P∆detr of 24.0 ± 3.6 cmH_2_O in males with NVC, respectively (Fig. 5d,e). The PVR was 155 ± 37 ml in males with voiding and significantly lower than a PVR of 246 ± 34 ml in males with NVC (*p* = 0.03) (Fig. 5f). The latter finding is consistent with the lack of emptying of bladder by voiding in the males with NVC. In addition, the Capacity-IV25 was 225.6 ± 36.9 ml in males with voiding and not significantly different from 245.7 ± 34.0 ml in male subjects with NVC. Qualitatively, the pressure profiles were different between the voiding and non-voiding groups. With onset of voiding, the bladder pressure typically decreased gradually. In contrast, in the setting of NVCs, the bladder pressure remained elevated for a longer interval.

## Discussion

Urodynamic investigations in the form of cystometry and pressure flow studies were performed under light ketamine sedation in adult rhesus macaques, and comparative analysis of data sets from both male and female subjects was performed. The studies established a comprehensive protocol for transurethral catheterization and demonstrated marked functional differences in LUT function between male and female rhesus macaques. Filling cystometry demonstrated significantly higher infused volume to raise the bladder pressure 25 cmH_2_O (IV25), compliance (Bcomp), and bladder capacity (Capacity-IV25) in the male compared to female subjects. Pressure flow studies showed activation of detrusor reflex contractions in all male and the vast majority of female subjects. However, voiding was shown in a significantly lower proportion of the male subjects compared to the females. Pressure flow studies showed a significantly higher bladder pressure at voiding onset, peak pressure, and elevation of the bladder pressure from the completion of bladder filling to the peak pressure in the male subjects, whereas there was no difference between male and female subjects in the delay to voiding after bladder filling, voided volume, voiding duration, voiding flow, and voiding efficiency. However, the PVR was significantly larger in the male subjects. Additional analysis of the male subjects with voiding or NVC showed no statistical difference between the groups in bladder pressure at the completion of bladder filling, IV25, compliance, Capacity-IV25, bladder pressure at the end of saline infusion, peak pressure, or in the increase in bladder pressure from the point of completion of bladder filling to the peak pressure. The PVR was significantly larger in the male cohort with NVC compared to the male cohort with demonstrated voiding.

All male and female subjects were adults and within the normal reproductive age range for rhesus macaques. There was no statistical difference in age between the two groups, but the male rhesus macaques showed a significantly higher body weight compared to the females, consistent with normal sexual dimorphism in size and weight of macaques^16,17^.

Ketamine has emerged as the preferred anesthetic agent for urodynamic recordings in rhesus macaques. Although all anesthetics suppress reflex function to various degrees in different species, ketamine has been found to better preserve the micturition reflex and detrusor contractions compared to several other agents, when it is administered to achieve a light plane of sedation^10,13–15,18^. However, ketamine is a dissociative anesthetic agent associated with relative reflex preservation and tendency for hypertonicity in skeletal muscles^19,20^. A ketamine-induced hypertonicity of pelvic floor muscles, including the external urethral sphincter (EUS), will further aggravate the technical challenges associated with transurethral catheter placement in male rhesus macaques. In the present studies, it was found that isoflurane sedation allowed for an easier placement of the transurethral catheter. Volatile anesthetic agents, including isoflurane, are commonly used and well tolerated in non-human primates, as they can be readily administered, their minimal alveolar concentration individually adjusted to achieve desired plane of anesthesia, and the subject emergence from anesthesia is rapid^21–23^. After successful catheter placement, the isoflurane was discontinued, allowed to be fully ventilated out from the alveolar space, and replaced by ketamine CRI administration for the urodynamic recordings in the present studies. There was no statistical difference between the urodynamic outcome measures tested between the male subgroups with or without prior isoflurane exposure for transurethral bladder catheter placement.

Potential limitations of the study are identified and related to e.g. the use of ketamine anesthesia, animal positioning, transurethral bladder catheterization, and varied rate of infusion for partial bladder filling between subjects. All anesthetic agents, including ketamine, are known to suppress reflex function, and is a likely contributor to e.g. the observed prolonged voiding period, low flow rate, and incomplete bladder emptying. However, the plane of ketamine anesthesia was kept at the same minimal level to achieve light sedation and immobilization in both the male and female subjects, so any relative differences in urodynamic outcome measures between the sexes would less likely be attributed to the anesthesia. The cystometrogram studies would therefore remain interpretable for the objectives of the present investigation. Due to technical aspects for ease of bladder catheterization, the female subjects were placed in prone position with hips and knees flexed to allow for access to the external urethral opening and to elevate the abdomen above the surface of the procedure table. The males were placed in a reclined sitting position to allow for straightening of the S-shaped urethra during catheter placement. The subjects were maintained in the original positions for catheter placement during the urodynamic recordings. Due to the potential differences in recording conditions from varied animal positioning, standardization of this technical aspect for both male and female animals will be a goal for future studies. Transurethral catheter placement may have contributed to a relative obstruction of flow during voiding, and it is possible that a relative obstruction to voiding may be more marked in males, as the male urethra is longer and has an S-shaped curvature that may reduce flow. This difference between the sexes may have contributed to a higher flow resistance and more frequent NVCs in males. The bladder filling was performed manually and showed individual rate differences, including an approximately 20% higher infusion rate in the female subjects. Such differences may have affected passive properties of the bladder, including compliance. However, the primary goal of a 25 cm H_2_O elevation of bladder pressure from the partial filling as well as reflex responses to bladder filling, including the delay to voiding and voiding duration were not different between the sexes. However, for standardization purposes and future studies, the transition to the use of an automatic pump with a pre-set infusion rate will be a goal.

In human urodynamic studies, bladder compliance is performed with an awake subject as an interactive procedure and calculated as a volume change divided by a bladder pressure change between two time points, typically after emptying of the bladder and after filling the bladder to elicit a sensation of first bladder filling or maximum capacity before voiding^24^. For urodynamic studies in non-human primates under sedation, compliance calculations will need to be modified but maintain volume and pressure measurements at two time points during a bladder filling procedure. We determined volume and bladder pressures after emptying of the bladder and at partial filling of the bladder at an aimed increase in bladder pressure to about 25 cmH_2_O above the baseline pressure in each subject.

Studies of bladder capacity also needed to be adapted for the rhesus macaque model. During human cystometrogram studies, subjects report first sensation of filling (FSF), first desire to void FDV), and strong desire to void (SDV), and the maximum cystometric capacity is calculated as the sum of the volume capacity for SDV and the post-void residual (PVR)^25^. Such subject feedback is not possible in animal studies, and maximum cystometric capacity may instead be obtained based on the bladder volume at the onset of voiding during continuous bladder infusion. However, it was opted not to infuse these subjects until voiding in the present study, as rhesus macaques have shown a relative refractory period with decreased detrusor contractility from repeated bladder fillings^26^. Instead, the infused volume to raise the bladder pressure 25 cmH_2_O served as a physiologic stimulus to evoke a delayed micturition reflex and voiding. The infused volume to raise the bladder pressure 25 cmH_2_O and the combined voided volume and PVR instead served as surrogate markers for bladder capacity assessments.

Functional differences were identified in both passive and active properties of the detrusor muscle between male and female rhesus macaques. Our findings included a significantly larger infusion volume to raise the bladder pressure 25 cmH_2_O (IV25) in the male subjects. A similar larger bladder capacity in men compared to women has been reported in human cystometry studies on maximum bladder capacity^27^. In more recent studies, however, men showed a larger bladder capacity at first sensation of filling^25,28,29^, but there was no difference between men and women for maximum cystometric capacity^25^. The present investigation also identified a higher bladder compliance in the male rhesus macaques. In clinical studies, a higher compliance was detected in female healthy volunteers compared to males^27^, whereas a subsequent clinical research study showed no difference in bladder compliance between men and women^25^.

Voiding is normally brief, and behavioral bladder emptying studies show an almost constant duration of 21 s across awake mammals over 3 kg with the urethra being suggested as serving as a flow-enhancing device^30^. In the present study, the evoked voiding phase was generally prolonged over several minutes and the bladder emptying was incomplete. The differences between the studies are likely multifactorial and include different experimental conditions. First, the present experiments were performed under anesthesia. Although ketamine is relatively reflex-sparing in rhesus macaques, all anesthetic agents suppress spinal reflexes to various degrees. Second, reflex voiding occurred as an evoked response to partial bladder filling and relied on both spinal and supraspinal reflex activation. Third, a multi-lumen transurethral bladder catheter was placed and provided partial obstruction to urine flow. Fourth, as a dissociative anesthetic, ketamine may preserve or increase the contractility of skeletal muscles, including the external urethral sphincter, thereby contributing to an increased urethral resistance to urine flow.

Pressure flow studies showed in the male rhesus macaques compared to the female subjects a significantly higher bladder pressure at onset of voiding, peak pressure, and detrusor-activated increase in bladder pressure after partial bladder filling. Direct comparisons between normal LUT function in men and women are relatively sparse, but differences in detrusor function have been reported between males and females based on urodynamic studies in human subjects. For instance, pressure flow studies in subjects without any known urological pathology have also indicated a lower detrusor contractility in women compared to men and a higher peak detrusor pressure and higher detrusor pressure during maximum flow rate in men compared to women^25,31,32^. A higher maximum detrusor pressure was also maintained in men compared to women with a spinal cord injury and undergoing sacral anterior root stimulation to facilitate voiding^33^. Animal studies also provide support to the notion of sexual dimorphism of detrusor function, as a higher maximum flow rate and longer micturition time were shown in male compared to female rats undergoing cystometry^34^. Male rats also demonstrated a higher bladder peak pressure and larger pressure decrease during voiding compared to corresponding recordings in female rats^35^.

Differences between males and females with regards to detrusor contractility and bladder pressures during micturition have been attributed to a greater outflow resistance in males^27,33,36^. The striated EUS muscle is well developed in male but not female rats^35^. The EUS also shows sexual dimorphism in rhesus macaques, as most of the male urethra is surrounded by a horse-shoe shaped sphincter except for a circular sphincter at the cranial third of the urethra, whereas the female urethra is surrounded by a circular sphincter, which surrounds both the urethra and vagina at the lower third of the urethra^37^. The notion of a greater outflow resistance in male rhesus macaques is speculative, however, as longer urethras have been suggested to provide higher gravitational force and higher flow speed^30^. The addition of EUS EMG recordings in future studies may assist in determining the functional role of the urethra during voiding in rhesus macaques.

In summary, the studies show feasibility and utility of comprehensive urodynamic investigations in both male and female rhesus macaques. Sexual dimorphism of detrusor function is demonstrated by filling cystometry and pressure flow studies with significant differences detected in both passive and active detrusor properties between male and female subjects. The pattern of the sex-related dimorphism of micturition reflexes in rhesus macaques is reminiscent of established differences in LUT function between men and women. Sexual dimorphism in LUT function needs to be taken into consideration in the design and implementation of translational research studies for the evaluation of e.g. disease processes, effects of neurological trauma, and treatment interventions. Rhesus macaques may therefore be considered a suitable model for pre-clinical studies in human disease models.

## Methods

All animal procedures and collection of physiological data were performed at the California National Primate Research Center (CNPRC), University of California at Davis, an academic institution accredited by the Association for Assessment and Accreditation of Laboratory Animal Care (AAALAC) International. All animal study protocols procedures were approved by the UC Davis Institutional Animal Care and Use Committee (IACUC). All animal procedures and care were also performed in compliance with the *Guide for the Care and Use of Laboratory Animals* provided by the Institute for Laboratory Animal Research^38^.

### Animals

A total of 38 male and female rhesus macaques *(Macaca mulatta)* were included as research subjects in the studies. The male animals were 7.3 ± 0.4 years old (n = 16) and the female subjects were 8.4 ± 0.6 years of age (n = 22). There was no statistical difference in age between the groups. However, the male subjects showed a higher body weight of 11.8 ± 0.7 kg compared to a body weight of 8.1 ± 0.3 kg in females (*p* < 0.0001). All subjects were adults and within the normal reproductive age, but phase of ovarian cycle was not determined. Both nulliparous females and animals with prior pregnancy and birth history were included. The female subjects had a history of 1.23 ± 0.44 pregnancies and 1.00 ± 0.39 live births (n = 22). Before study inclusion, all subjects were sedated by an intramuscular (IM) injection of ketamine (10 mg/kg) and underwent a physical examination by a CNPRC veterinarian to assess for physical fitness for study participation. Clinical laboratory screening, including a complete blood count with differential and a comprehensive metabolic panel, was performed to screen for any infectious, inflammatory, or metabolic conditions. All subjects entering the study were in good physical health and without signs of any neurological or metabolic condition.

### Urodynamic studies

Each subject was initially sedated by an intramuscular (IM) injection of ketamine (10 mg/kg) followed by the placement of an intravenous (IV) catheter for fluid and anesthetic administration and an endotracheal tube for airway protection. Ketamine was next administered at 10–12 mg/kg/hour IV by constant rate infusion (CRI), and the infusion rate was adjusted individually to achieve light sedation and immobilization. For urodynamic studies, a triple-lumen 7-Fr transurethral bladder catheter or a double lumen 6-Fr transurethral catheter (Laborie Medical Technologies, Corp, Williston, VT) was placed. This was performed with female subjects in prone position with flexion at hips and knees and with males in a reclined sitting position to allow optimal access for transurethral bladder catheter placement. The animals were maintained in the same position for the urodynamic studies. In a subset of male animals (n = 8), the transurethral bladder catheter was placed under a surgical plane of isoflurane anesthesia to allow for sufficient relaxation of the EUS muscle to facilitate catheter passage through the portion of the urethra surrounded by the sphincter. Following catheter placement, the isoflurane was discontinued, ventilated off, and all subjects were maintained under a light plane of ketamine by CRI with the infusion rate adjusted individually to achieve sedation and immobilization at the lightest possible plane of anesthesia. No recordings were initiated until the minimum alveolar concentration (MAC) of isoflurane was zero. To investigate whether isoflurane may have caused any delayed effects on micturition reflexes, urodynamic outcome measures, including bladder capacity, bladder compliance, peak pressure during micturition, and post-void residual volume, were compared between sub-groups of males with isoflurane exposure (n = 8) and those without isoflurane administration (n = 8). There was no statistical difference for the urodynamic outcome measures between the two sub-groups, so they were merged into one male cohort (n = 16) for the urodynamic studies.

For cystometrogram (CMG) recordings, the cystometry port of the catheter was attached to a TSD 104A pressure transducer system and connected to an MP 150 Data Acquisition System (Biopac Systems, Goleta, CA). Next, the bladder was emptied and subsequently partially filled with saline at room temperature, using the fill port of the triple or double lumen catheter. Manual saline infusion was performed at a calculated rate of 71 ± 5 ml/min in the males (n = 16) and at 85 ± 4 ml/min in the female subjects (n = 22). The bladder pressure was monitored continuously and increased by the bladder filling from a baseline pressure of 1.3 ± 1.1 cmH_2_O in the males and 0.2 ± 0.6 cmH_2_O in the females with the goal of reaching a target bladder pressure of about 25 cmH_2_O above the baseline pressure to act as a stimulus for the micturition reflex. No voiding was observed at the end of the partial bladder filling. Instead, a subsequent delayed reflex detrusor contraction resulted in a gradual further increase of the bladder pressure and voiding. CMG recordings documented the bladder pressures associated with bladder filling, onset of the micturition reflex, and voiding. A total of 2–3 cycles of partial bladder filling were performed in an attempt to activate detrusor contractions, and the first reflex cycle with voiding contractions was used for analysis. The first successful voiding contraction was used as repeat voiding cycles may cause a compromised detrusor response in the absence of an extended recovery period between bladder fillings^26^. In a subset of animals, the partial bladder filling procedure resulted in non-voiding (NV) or in non-voiding contractions (NVC). The NVC were defined as a delayed activation of the micturition reflex with an increase of bladder pressure of at least 10 cmH_2_O above the bladder pressure at the end of bladder filling but without any demonstrated voiding. All subjects recovered well from the anesthesia and returned to normal baseline activities after the completion of the CMG procedures.

The urodynamic studies provided a comprehensive set of quantitative outcome measures, including baseline bladder pressure after bladder emptying (Pbase), bladder pressure at the completion of partial bladder filling with saline (Pfill), change in bladder pressure from partial bladder filling (P∆fill), duration of partial bladder filling by saline infusion (Fill Dur), infusion rate of partial bladder filling with saline (Fill Rate), infused volume of saline to increase bladder pressure about 25 cmH_2_O above Pbase (IV25), bladder compliance calculated as IV25/P∆fill (Bcomp), time between end of bladder infusion and onset of reflex voiding (Vdelay), bladder pressure at onset of voiding (Pvoid), peak pressure during detrusor contraction (Ppeak), increase in pressure from end of bladder filling to peak pressure during detrusor contraction calculated as Ppeak–Pfill (P∆detr), duration of voiding (Vdur), voided volume (Vv), flow rate of voiding calculated as Vv/Vdur (Vflow), voiding efficiency calculated as Vv/infused bladder volume (VE), post-void residual volume determined by withdrawal of bladder contents after voiding (PVR). Capacity-IV25 was calculated as the sum of the voided volume and PVR following partial filling of an emptied bladder to a volume that resulted in an increase in bladder pressure of 25 cmH_2_O.

### Statistical analysis

All data are presented as mean ± standard error (SE). The N-1 Chi-squared test was performed for comparing two proportions using the MedCalc Comparison of Proportions Calculator (MedCalc Software, Ostend, Belgium). We used GraphPad Prism, version 7.05, (GraphPad Software, Inc, La Jolla, CA) to first test the data for normal distribution followed by t-test studies or the non-parametric Mann Whitney U-Test to compare data sets between groups. A value of *p* < 0.05 was considered to reflect a statistically significant difference between groups.

## Data Availability

The datasets generated during and/or analyzed during the current study are available from the corresponding author on reasonable request.
